# Electrical Properties of Carbon Nanotubes: From Individual to Assemblies

**DOI:** 10.3390/nano15151165

**Published:** 2025-07-28

**Authors:** Yuxin Xiang, Lili Zhang, Chang Liu

**Affiliations:** Shenyang National Laboratory for Materials Science, Institute of Metal Research (IMR), Chinese Academy of Sciences, 72 Wenhua Road, Shenyang 110016, China; yxxiang22s@imr.ac.cn

**Keywords:** carbon nanotubes, assembly, electrical property, measurements

## Abstract

Carbon nanotubes (CNTs) have attracted intense research interest owing to their unique one-dimensional structure and exceptional properties. However, when individual CNTs are assembled to macrostructures such as films and fibers, their electrical performance often deteriorates significantly. This review offers a comprehensive look at the recent progress in the electrical properties and measurement techniques of CNTs, ranging from individual nanotubes to their assemblies. Firstly, we explore the methods for measuring the electrical properties of individual CNTs, including scanning tunnelling microscopy, electron microscope-based nanoprobes, and measurements using nanodevices. Secondly, we examine how structural characteristics of CNTs (e.g., chirality, diameter, and defects) influence their electrical behaviors. A critical comparison between individual CNTs and their assemblies reveals the difficulties in transferring the electrical properties from nanoscale to bulk materials. Finally, we put forward strategies to boost the electrical conductivity of CNT assemblies and also sketch out future research and development directions.

## 1. Introduction

**Figure 1:**
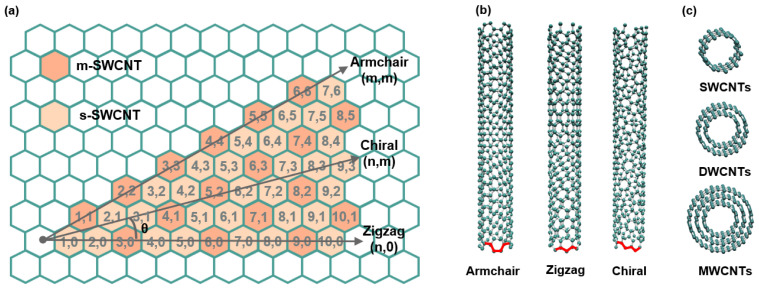
(**a**) Schematic diagram of an unrolled SWCNT with chiral vectors and two basis vectors. (**b**) “Ball and stick” models of (5,5) armchair SWCNT, (8,0) zigzag SWCNT, and (8,5) chiral SWCNT (from left to right). (**c**) “Ball and stick” models of SWCNT, DWCNT, and MWCNT.

With the progress of science and technology, there is ever-increasing demand for various electronic devices in the fields of information, display, and sensing. These demands include high-performance, miniaturization, and flexible wearable devices, among others. They present substantial challenges to materials and device design. Since the 1980s, scientists have successively discovered a series of low-dimensional materials such as quantum dots, nanotubes, nanowires, graphene, and graphdiyne. These materials have unique structures and extraordinary properties. It is expected that they will meet the requirements of the post-Moore era in terms of multi-functionality and miniaturization. Among them, carbon nanotubes (CNTs), reported by Sumio Iijima in 1991 [1], are a promising material for achieving large-scale practical applications and taking the lead in future competition within the high-tech industry. CNTs are one-dimensional hollow tubular structures made of sp^2^-hybridized carbon atoms. More specifically, single-walled CNTs (SWCNTs) are uniquely defined by chiral index (n, m), since they can be taken as rolled graphene sheets at different chiral angles θ, corresponding to specific chiral vectors ***C*** = n·$a_{1}$+ m·$a_{2}$, where $a_{1}\;=\;a(\sqrt{3},0)$ and $a_{2}\;=\;a(\frac{\sqrt{3}}{2},\frac{3}{2})$ are the basis vectors in graphene lattice (Figure 1a) [2]. As is shown in Figure 1b, SWCNTs will be referred to as zigzag SWCNTs if m = 0, while armchair type if m = n and chiral SWCNTs if n ≠ m. SWCNTs are then categorized electrically into metallic SWCNTs (m-SWCNTs) when (n − m)/3 = q (an integer); otherwise, they are referred to semiconducting SWCNTs (s-SWCNTs) [3]. Depending on their wall number, CNTs are classified as SWCNTs, double-walled (DWCNTs), or multi-walled (MWCNTs) (Figure 1c).

In general, CNTs possess extraordinary and tunable electrical properties, making them promising candidates for use in transistors, energy storage, transparent conductive films, and other electrical applications [2,4]. A 10 nm long CNT could carry current up to 70 μA [5]. An SWCNT has been demonstrated to withstand a current density of 10^9^ A/cm^2^ [6], 2–3 orders higher than copper wires. The intrinsic carrier mobility of an intact s-SWCNT can reach 10^5^ cm^2^ V^−1^ s^−1^ [7,8] at room temperature, which is 100 times higher than that of bulk phase silicon with the dopant concentration of 10^17^ cm^−3^ [9]. Furthermore, adjusting the gate voltage of a field-effect transistor (FET) enables the concentration and type of the carriers to be regulated, thereby producing either p-type or n-type semiconductors.

CNTs possess extraordinary ballistic transport characteristics. When the length of a CNT is shorter than the mean free path of electrons, which can extend to approximately a micron [9,10], electron scattering becomes negligible. In this case, the CNTs exhibit high current-carrying capability (~25 μA per tube) and the length of the CNT is independent of its resistance, in line with the law of ballistic transport [6,11]. Nevertheless, ballistic transport can only be maintained when a low bias voltage (0.1 V ≤ |V_ap_| ≤ 6 V) is applied to the CNT device; otherwise, scattering between electrons and phonons is significant. When a low bias voltage is applied to a CNT device with a length of several hundred nanometers, the carrier can exhibit ballistic transport characteristics [12].

Under the ideal condition of ballistic transport, the conductance of an SWCNT with two conduction channels is equal to $\frac{4e^{2}}{h}\approx 77.5\;\mu S$, i.e., a resistance of 6.45 kΩ [13,14], which is a temperature-independent minimum resistance (this value being about 10^7^ higher that clean Cu/Cu contacts). If an electrode with appropriate work function is selected, the on-state conductance of the s-SWCNT device can also be close to $\frac{4e^{2}}{h}$ [1,2,3]. When the length of SWCNT continues to increase to several microns, reaching the regime of scattering-dominated electrical conduction, the resistance undergoes a persistent decline.

However, the electrical properties of individual CNTs, as measured experimentally, are much lower than predicted due to the existence of defects and impurities. These properties continually diminish significantly in assemblies due to the introduction of more interfaces and the diverse structures of the comprising CNTs. In other words, the electrical properties of CNTs depend on various structural features, such as chirality, length, defects, and the configuration of CNT assemblies. For example, the highest measured carrier mobility of SWCNT film is about 10^3^ cm^2^/(V·s) [15], and the highest measured conductivity of SWCNT fibers is about 10^7^ S/m [16,17], which are much lower than those of individual SWCNTs. Recent advancements in flexible electronics and wearable sensors have spurred increased efforts to optimize CNT assemblies. However, it remains a big challenge to achieve the excellent performance of CNTs at macroscopic scale.

**Figure 2:**
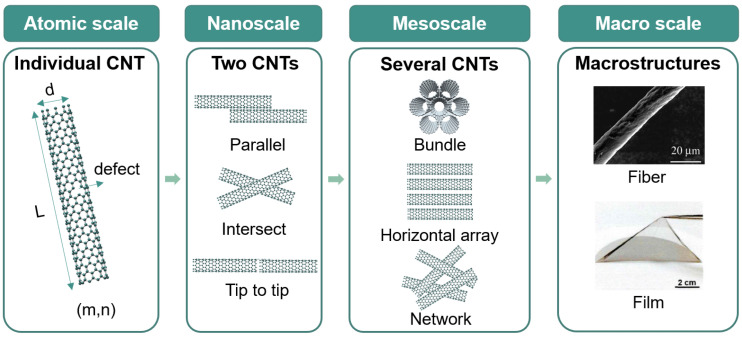
Schematic showing the structures of individual CNTs and their assemblies at different scales.

In this review, we present an overview of the electrical properties of CNTs from the individual level to macroscopic assemblies, and how these properties are measured. We begin by examining the methods used to measure the electrical properties of CNTs, including scanning tunnelling microscopy (STM), electron microscope-based nanoprobes, and nanodevice-based measurements. The origins of measurement error will be discussed. Next, we summarize the effects of structural features affecting the electrical properties of individual CNTs, CNT bundles, and macroscopic assemblies (Figure 2). It should be noted that the bundles in this review are those comprising countable CNTs with a diameter of 50 nm or less. The macroscopic assemblies of CNTs refer to vertical arrays, aerogel, soot, films, and fibers. The scope of this review is limited to the most widely used assemblies, namely, films and fibers. Furthermore, attention will be paid to the mechanisms underlying the difference in the electrical properties between individual and assembled SWCNTs. Finally, we discuss possible solutions such as doping to improving the electrical properties of individual CNTs and CNT assemblies. Future trends toward bridging the gap between the electrical properties of individual and assembled CNTs are also outlined.

## 2. Measurement of the Electrical Properties of CNTs

### 2.1. Methods for Measuring the Electrical Properties of CNTs

This section summarizes three typical methodologies for measuring the electrical properties of CNTs: (1) STM, (2) electron microscope-based nanoprobes, and (3) nanodevice-based measurements. These methods can provide critical information on various electrical properties, including the density of states and resistance. Additionally, experimental errors and their potential sources are discussed.

#### 2.1.1. STM

**Figure 3:**
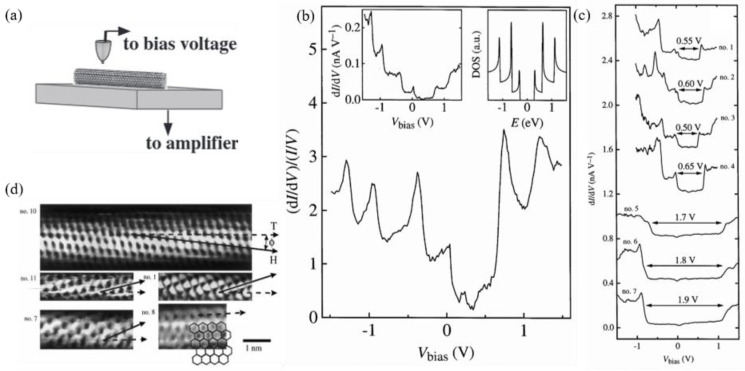
(**a**) Schematic diagram of the electrical measurement of individual SWCNTs using STM [19]; (**b**) STS diagram (local DOS at surface via tunneling) and DOS diagram [18]; (**c**) differential conductance (dI/dV) diagram [18]; (**d**) STM image of a SWCNT with atomic resolution [18].

STM is commonly used to characterize the surface structure of CNTs with atomic resolution and to obtain the surface electron state density through scanning tunneling spectroscopy (STS). Figure 3a schematically shows the working mechanism of an STM. A direct current with an additional small alternating voltage is applied between a CNT and a probe. The differential conductance (dI/dV) can then be calculated from tunnelling current versus voltage (I-V) data, which is considered to be proportional to the density of states (DOS) of the examined CNT (Figure 3b) [18]. When the gap value in the differential conductance diagram is 0.5–0.6 V or 1.7–2.0 V, the SWCNT is semiconducting or metallic, respectively [18]. Some differential conductance diagrams of SWCNTs are displayed in Figure 3c.

Atomically resolved STM images of individual SWCNTs can also be obtained using the STM technique (Figure 3d). Only a few carbon atoms at the top of the CNT are discernible. It is sufficient to enable the chirality to be identified unambiguously from the chiral angle θ.

#### 2.1.2. Electron Microscope-Based Nanoprobe Measurement

Using an electron microscope-based nanoprobe, it is possible to manipulate, process, and measure samples in situ in a scanning electron microscope (SEM) or a transmission electron microscope (TEM). Data relating to current voltage, current time, temperature profile, and power time can be obtained in conjunction with a series of SEM/TEM images. This makes it possible to establish a correlation between the microstructures and electrical properties of CNTs.

A nanoprobe can manipulate a sample or a nanoprobe electrode, triggering movement in the X, Y, and Z directions at the nanometer scale. In order to manipulate nanomaterials, the probe that grabs the sample must be sharp enough, with the tip size within 100 nm. Compared with mechanical methods of probe fabrication, such as polishing and shearing, electrochemical etching is the most common method due to its good reproducibility and standardization. By finely tuning the applied electrochemical potential, cutoff voltage, etching mode, electrolyte type and concentration, immersion depth, counter electrode type, and probe material, uniform and sharp probes with fewer impurities can be produced [20,21]. A variety of materials have been used for the nanoprobes. The most commonly used materials include tungsten [22,23,24,25,26,27,28,29,30,31] with high hardness and gold [32,33,34,35,36,37,38,39,40,41] with high chemical stability and good wettability.

Contact resistance between the nanoprobe and CNTs is a key concern in this type of nanoprobe measurement. To minimize its impact on the measured resistance of CNTs, the contact points between the tip and the CNT were irradiated with a focused ion beam (FIB) [42] or heated with a pulsed current [43]. Since CNTs can be welded to the wire to form good physical and electrical contacts, electrical contacts can also be formed by filling the tube with a material that has low vapor pressure, such as a chalcogenide [27], or by depositing nanoparticles that have a low melting point, such as tin [44,45].

**Figure 4:**
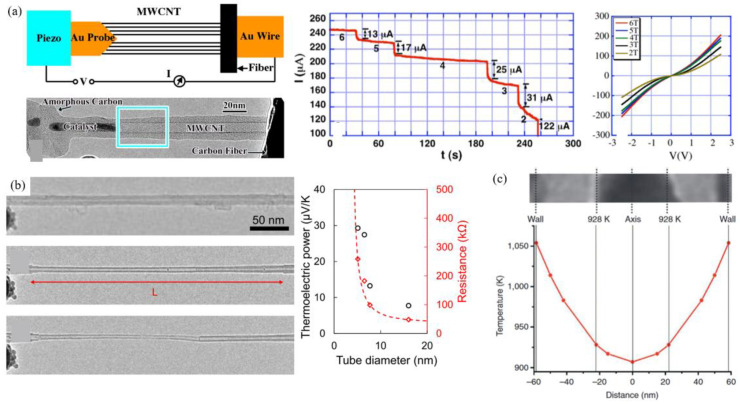
(**a**) Schematic of the nanoprobe measurement platform, TEM image of the connection, I-t curve of the breakdown of an MWCNT, and I-V plots with a different number of walls [39]. (**b**) Thermoelectric power and resistance measured during the step-by-step breakdown of an MWCNT wall [44]. (**c**) Estimation of the radial temperature profile in an electrically heated CNT channel [27].

Since SWCNTs have much lower structure stability and are more difficult to manipulate under an electron microscope, the nanoprobe technique has mainly been used to investigate the electrical properties of individual MWCNTs. For instance, Huang et al. [39] reported unexpectedly three distinct breakdown sequences in individual MWCNTs, proving that each wall of an MWCNT conducts at high voltage, since a current drop can be observed when the innermost wall was broken. Moreover, the breakdown of each wall initiated in the middle of the tube, proving the transport property is not ballistic (Figure 4a). Also, the thermoelectric power increased as the tube diameter decreased (Figure 4b). Another crucial phenomenon that can be observed is the structure engineering by Joule heating. On exposure to a high current density, resistive hotspots near the contact points later migrated and expanded along the CNT over time, as indicated by the localized sublimation of the encapsulated material (Figure 4c) [27]. The electrical properties of doping systems can also be investigated by this method. It has been observed by Aslam et al. [28] that a large current could induce structural transformation of N-doped MWCNTs, resulting in the removal of the dopant, and a significant change in electrical behavior.

As a conclusion, using this method with SEM/TEM enables more phenomena relating to the Joule heating behavior of CNTs and the doping system to be observed, particularly with regard to the CNT filling system and electromigration processes.

#### 2.1.3. CNT Electronic Devices

A great deal of work has been carried out on designing electronic devices using individual CNTs, CNT arrays, and CNT films, as they are ideal for use in integrated circuits (ICs). One common device is the four-point probe measurement [24,46,47,48,49]. This device-based method is often used to measure the electrical resistance of individual CNTs [24,46,47,48], CNT fibers [15,16,49,50], and CNT films. This can be achieved by passing a current through the two outer terminals (I_outer_) and measuring the voltage using the two inner terminals (ΔV_inner_). All electrodes must be in contact with the material being measured. This method can mitigate contact resistance errors, and reveal the material’s intrinsic resistance, which can be determined by R = (ΔV_inner_)/I_outer_ [51]. Furthermore, numerous methodologies can be employed to reduce contact resistance between the electrodes and CNTs. Ebbesen et al. [46] reported on four-probe measurements of individual SWCNTs produced by the ion-induced deposition of four 80 nm wide tungsten leads. Wang et al. [52] measured the electrical resistance of 5 cm long single CNT fiber by dipping silver adhesives to four contact regions.

Another widespread electronic device is the CNT-based FET. Due to their ultrahigh carrier mobility and satisfactory carrier concentration, CNTs are a promising material for the fabrication of future ICs. A CNT-based FET can inhibit the short-channel effect in a conventional metal–oxide–semiconductor FET (MOSFET) and can be scaled to a contacted gate pitch of 55 nm, corresponding to a sub-10 nm node. The carrier mobility (1500 cm^2^ V^−1^ s^−1^) and Fermi velocity (12 × 10^6^ cm s^−1^) of this node are higher than those of a 10 nm MOSFET [53]. Also, a CNT-based FET could achieve a remarkable reduction in subthreshold swing (Ss) to ~35 mV/dec, which would significantly reduce power consumption [53,54]. Ultrahigh carrier mobility directly enhances the transconductance (g_m_) and cutoff frequency (f_T_), exhibiting favorable high-frequency performance [55]. These parameters are of primary concern in the future development of ICs, and these technological advancements are expected to promote the practical application of carbon-based circuits [56].

**Figure 5:**
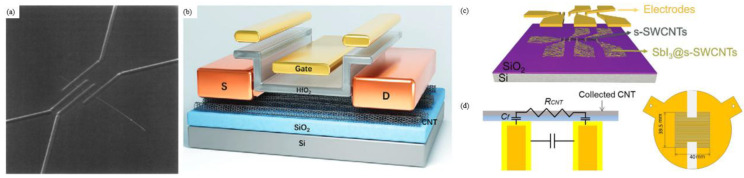
(**a**) FIB image of the four-probe measurement on an individual CNT [46]; (**b**) schematic diagram of a top-gate CNT-based FET [65]; (**c**) schematic diagrams of a thin film transistor device [54]; (**d**) cross-sectional diagrams and partial view of the in situ measurement device placed under a collection filter during CNT collection [73].

The CNT-FET structure comprises five parts. The channels are made of CNTs, either in the form of individual s-SWCNTs (Figure 5a) [11,57], an aligned s-SWCNT array (Figure 5b) [58,59], or a thin film (Figure 5c) [54,60,61,62]. The source and drain electrodes are made of metals with a high work function, such as palladium [53,63] and platinum [64]. The gate electrode can be either top-gate [65] or back-gate [65,66]. The high-k gate dielectric can be HfO_2_ [56,65,67,68], Al_2_O_3_ [64,69], or ultrathin SiO_2_ [70]. The insulating substrate can be SiO_2_/Si, sapphire [71], or hBN [72], or a flexible organic substrate.

Some special electronic design schemes are also presented to facilitate research into the structure–performance relationship. For example, Oshima et al. [73] proposed an in-situ measurement of the electrical impedance during CNT synthesis by floating catalyst chemical vapor deposition (FCCVD) (see Figure 5d) to monitor CNT film thickness and reveal its correlation with the percolation threshold.

### 2.2. Measurement Errors and Their Sources

The previously mentioned methodologies can provide a variety of electrical information, such as band gap, resistance, and thermoelectric power. These approaches have one thing in common: the necessity of establishing contact between CNT and electrodes. In addition to contact resistance, other factors have also been identified as contributors to the diversity of electrical measurement results in different experimental groups. These include variations in measurement environment (such as gas composition, temperature, and humidity) and substrate. Concurrently, a variety of macrostructures with distinct intrinsic and configuration-specific CNT arrangements manifest diverse array electrical properties. These issues pose a significant challenge when it comes to measuring electrical properties.

The primary challenge lies in minimizing the contact resistance. We then model the resistance of an individual CNT contacted at each end of the metal electrodes as the sum of the three parts (Formula (1)).

**Figure 6:**
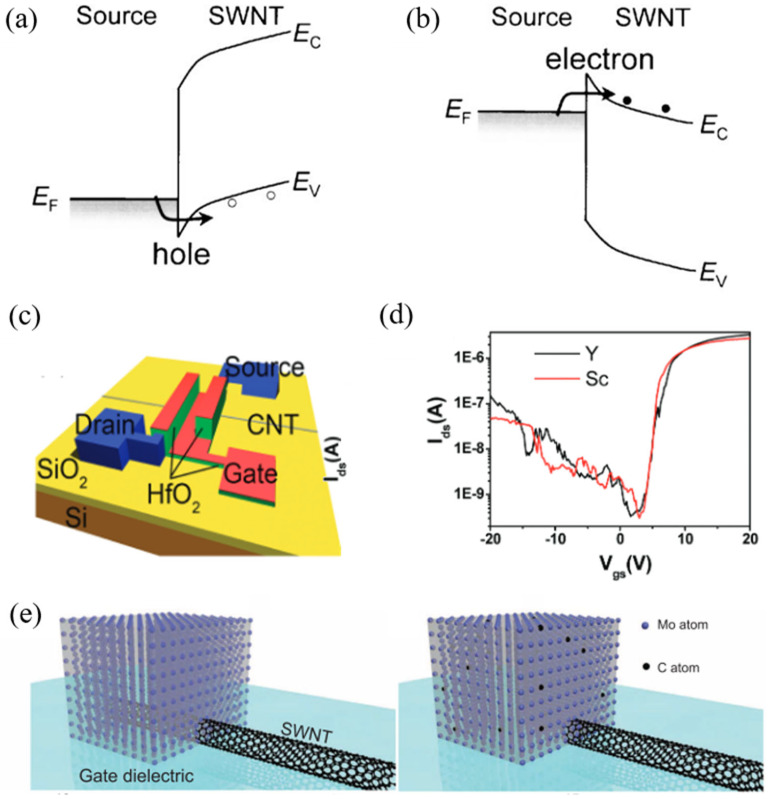
Band structure of the contact interface between s-SWCNT and metal electrodes with a (**a**) high work function and (**b**) low work function [80]; (**c**) Y-contacted top-gate CNT-FET device and (**d**) its performance compared to Sc-contacted device; (**e**) schematics showing the conversion from side-bonded contact to end-bonded contact with Mo in SWCNT transistors, and the transfer characteristics of typical Mo end-contacted SWCNT transistors [77].

R_CNT_ = R_Q_ + R_L_ + R_CONTACT_ (1)

where R_Q_ is called the quantum resistance. For an m-SWCNT with ideal ballistic transport and perfect contacts, R_Q_ ≈ 6.5 kΩ, which is the minimum theoretical resistance per conduction channel in a 1D system and fundamental limit from 1D quantum transport. This is owing to the inevitable mismatch in the number of conduction channels between the nanotube and the macroscopic metal electrode [74]. R_L_ is the scattering resistance due to electron–phonon scattering, impurities, and defects. If the nanotube length is shorter than the mean free path, which is about 300–1000 nm, we can usually neglect R_L_. R_CONTACT_ is the most variable and often dominant component in experiments, which depends on work function difference between the metal and the CNT (the work function of SWCNT is about 4.8 eV [75]), metal wettability, and the bonding configuration [76] (e.g., Pd makes better contacts than Au) and contact geometry (end-bonded vs. side contact) [77]. R_CONTACT_ is about 5–50 kΩ with Pd [78]. As a result, the choice of metal electrode is crucial for reducing contact resistance. For metal electrodes with high work functions, their Fermi levels are close to the valence band of s-SWCNTs. This means holes are more easily injected into the channel than electrons, behaving as p-type semiconductors (Figure 6). Pt, Au, Pd, and Rh [79] are the electrodes of wide use in this category, among which Pd is the most suitable precious metal with a good wettability [11].

By contrast, achieving n-type ohmic contact encounters more difficulties, as metals with relatively low work functions tend to oxidize first rather than react with carbon. However, it is still possible to realize end-bonded n-type contacts even with high work function metals through electrostatic doping near the source electrode [81,82]. Moreover, the Fermi level of Al, Sc, and Y is close to the conduction band of s-SWCNTs, also behaving as n-type [78,83,84]. Unlike forming p-type ohmic contact between Pd and CNT, Al can only form Schottky contact with a high barrier [78]. Regarding the economic benefit, Y is the most potential electrode in large-scale CNT ICs [84].

Except for the metal electrodes, graphene [85], hybrid graphene–metal [86], and CNT film [15,87] can also be considered as the electrode material. They can achieve significantly lower resistance than conventional metal electrodes, making them highly beneficial for advanced electrode applications. This stems from graphene’s exceptional conductivity, synergistic effects in hybrids or CNTs acting as conductive bridges that enhance charge transfer. These materials also offer superior flexibility, corrosion resistance, thermal stability, and higher surface area—enabling more efficient, durable, and miniaturized devices. Consequently, they outperform metals where low contact resistance, weight savings, or mechanical resilience are critical, though cost and fabrication complexity remain challenges for mass adoption [88].

Reducing contact resistance is essential to achieving desirable performance of FETs, such as high field-effect mobility and on/off ratio. A comparison on the methods for reducing the contact resistance at the individual CNT–electrode interface is shown in Table 1.

In addition to the selection of electrode materials, the strategies like chemisorption involving carbide formation through high-temperature annealing and local Joule heating with end-bonded type have also been demonstrated to reduce contact resistance. This could form contact with size-independent low resistance and high physical stability [28,29,77]. Such an all-shell connection is demonstrated to prevent the field-emission failure at the CNT–metal interface, promising for real-world applications in CNT-based electronic devices [30]. This chemisorption process has been shown to facilitate substantial interfacial charge transfer via the formation of chemical bonds. This, in turn, results in alterations to the electronic structure, enhancement of durability, and the facilitation of charge injection. The contrary process is known as physical adsorption, which is defined as the process of minimal charge redistribution through weak electrostatic forces, thereby preserving bulk properties and being reversible [93].

Although novel approaches such as ultrasonic nanowelding and electron beam-induced deposition are reliable and repeatable to form good electrical contact, they are hindered by scalability concerns. More efficient methods must therefore be developed.

One impact on the precision of electrical measurement is regulating the measurement environment, including the gas composition, temperature, and humidity. The electrical properties of CNTs have been shown to be highly sensitive to the measurement environment, making this a key consideration in the design of any CNT-based measurement systems. Exposure to air reversibly influences the electrical resistance of the individual SWCNTs, like the conversion from s-SWCNT to m-SWCNT and CNT networks by negligible adsorption of O_2_ gases [94]. This can be attributed to the hole-doped effect in the presence of adsorbed oxygen or work function change of the metal contact [95]. These results, although demonstrating the potentially viable application on sensitive chemical oxygen detectors [96,97,98], likewise indicate that many intrinsic properties measured on as-prepared tubes might be substantially compromised by exposure to the external environment. Temperature is also a significant factor. It has been demonstrated that m-SWCNTs exhibit a near-linear increase in the calculated resistivity with temperature over a wide range due to twiston scattering [99]. For s-SWCNT, the on current decreases slightly with rising temperature due to enhanced phonon scattering, while the off current increases significantly due to thermal emission over Schottky barriers. Meanwhile, threshold voltage shifts negatively with temperature, attributed to changes in carrier injection and trap dynamics [65]. The humidity effect is also an essential factor in electrical measurement, especially for flexible electronics applications. Mostafa et al. [100] demonstrated that an increase in relative humidity from 10% to 80% can result in a reduction of approximately 4% in the electrical conductivity of vertically aligned CNTs.

Another impact on the precision of electrical measurement in CNT devices is the substrate. In general, CNTs in FETs are in direct contact with the substrate, which gives rise to several non-ideal behaviors. The additional carriers will be trapped from the substrate, inducing the electrical noise by changing the surface potential [101]. A rough substrate would decrease the mobility and conductivity of the FET devices. Moreover, due to the attachment of CNT and the substrate, a part of the sensing area is lost [102]. As a result, it seems that FETs with suspended CNTs would exhibit higher electrical and sensitive performance. Some efforts were also made to change the type of substrate. Zhang et al. [72] reported on the direct growth of 2D close-packed SWCNT van der Waals crystals with uniform chirality and good alignment on a hexagonal boron nitride substrate. FETs constructed from these SWCNT arrays exhibited excellent electrical performance at room temperature. It demonstrated that atomically flat substrate could lead to a self-assembly growth of the low-dimensional material through van der Waals attraction and ultralow sliding friction.

## 3. Electrical Properties of CNTs

In this section, we discuss the electrical properties of individual CNTs, CNT bundles, and CNT assemblies. The structure-dependent electrical properties include quantitative results such as resistance, conductance, mobility, on/off ratio, and the density of state. Furthermore, the reasons why the electrical properties of individual CNTs do not translate into assemblies will be explained. Certain endeavors like doping to enhance the electrical characteristics of assemblies will also be discussed.

### 3.1. Electrical Properties of Intrinsic Structure of Individual CNTs

Individual CNTs are believed to exhibit superior electrical properties, which are strongly dependent on their intrinsic structures, including chirality, diameter, number of walls, defects, length, and doping levels.

#### 3.1.1. Chirality and Diameter 

**Figure 7:**
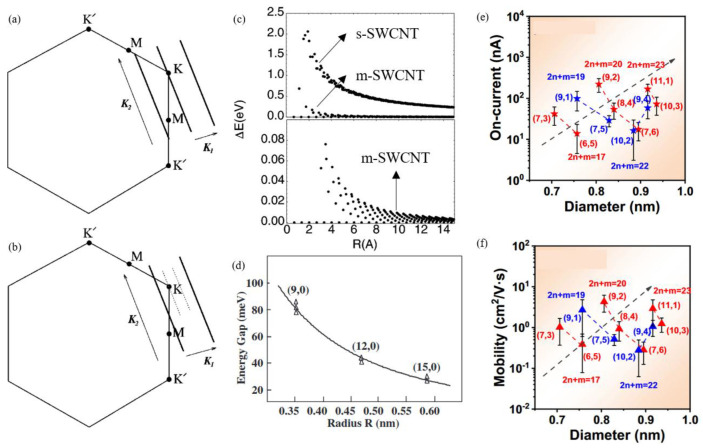
The wave vector k is shown as bold lines for (**a**) m-SWCNTs and (**b**) s-SWCNTs in the Brillouin zone of graphite (hexagon) [103]. (**c**) Gaps calculated for SWCNTs with a radius of less than 15 Å. Those with zero primary gap but nonzero curvature induced gaps which scale as 1/R^2^ are shown in the lower curve on top panel, whose expanded scale is shown in the lower panel [107]. (**d**) Curvature-induced gaps in “metallic” zigzag SWCNTs [108]. (**e**,**f**) Statistical distribution of the on-state current and the mobility of the two types. Blue and red symbols represent Type I and Type II, respectively.

The diameter and chirality of CNTs have been identified as the most crucial factors affecting its electrical properties. The one-dimensional dispersion relationship of electron bands in a SWCNT is calculated on the basis of the first principles of graphene band structure and local-density-functional approach. The results indicate that SWCNTs can be categorized into two distinct groups. When the rolling index n − m = 3q (q is an integer), the electron state wave vector k intersects at the primary Brillouin zone boundary. As demonstrated in Figure 7a, the conduction and valence bands intersect at the Fermi level, indicating that the SWCNT exhibits metallic characteristics with a zero-band gap. However, when (n − m)/3 ≠ q (q is an integer), the SWCNT behaves as a semiconductor (Figure 7b), and the band gap is inversely proportional to the tube diameter, as outlined in Formula (2) [103]. Consequently, the ratio of m-SWCNT to s-SWCNT is approximately 1:2 [104]. The occurrence of metallic behavior has been observed in armchair and select zigzag tubes, while semiconducting tubes have been shown to exhibit diameter-dependent band gaps (E_g_ ∝ 1/d).

(2) $$E_{\mathrm{gap}}=\frac{2\gamma_{0}a_{c‐c}}{d}$$

where $\gamma_{0}$ stands for the overlap energy of C-C bound with the value of about 347 kJ/mol, $a_{c‐c}$ denotes the nearest C-C bond length with the value of 0.142 nm, and d is the tube diameter [105].

However, it should be noted that there are exceptions to this rule in chiral and zigzag SWCNTs with very small diameters (d < 0.6 nm) due to curvature effects. It has been established that a portion of SWCNTs, predicted to be metallic, exhibits semiconducting properties [106]. The finite curvature reduces the overlap between adjacent p orbitals and shifts the band intersections away from the K point, resulting in a curvature-induced band gap in the metallic tube. Furthermore, the width of this band gap is inversely proportional to the square of the tube’s diameter [107] as shown in Figure 7c, which has been confirmed experimentally by Ouyang et al. (Figure 7d) [108]. Nonetheless, armchair SWCNTs are still metallic because k_F_ remains in the sub-band, with both the initial band gap and the curvature-induced band gap being zero [109].

Moreover, s-SWCNTs with small diameters can be divided into two types as shown in Figure 7e,f. For Type I (mod (2n + m, 3) = 1), both the on-state current and the carrier mobility exhibit an increase with an increasing chiral angle within the same family (2n + m = the same integer), whereas the results are the opposite for Type II (mod (2n + m, 3) = 2) [60].

Increases in diameter invariably result in greater numbers of walls being incorporated into current synthesis techniques. There are also a lot of electrical experiment results in MWCNTs [110]. The electrical properties of MWCNTs are inferior to those of SWCNTs, as theoretical explanations posit the occurrence of electronic distortions in MWCNTs, depending on how the lattices are interrelated [111]. In addition, experimental evidence was presented indicating that only the outermost shells contribute to the overall conduction at low bias. However, at high bias voltage, all of the shells contribute to carrier transport [104,105]. This leakage was frozen at a low temperature and low bias limit, indicating that the intershell coupling was thermally activated and tunnel-type [112]. As Liu et al. [113] also concluded in the context of DWCNTs, the only exception is the s/m-DWCNT (where the outer and inner layers are s-SWCNT and m-SWCNT, respectively), which only exhibits semiconducting-like properties and a much lower on/off ratio.

#### 3.1.2. Defect

Defects in CNTs, such as vacancies and non-hexagonal rings, which may be induced by irradiation or strain, can drastically change their transport properties. This section will discuss how and why the defects influence the electrical properties.

**Figure 8:**
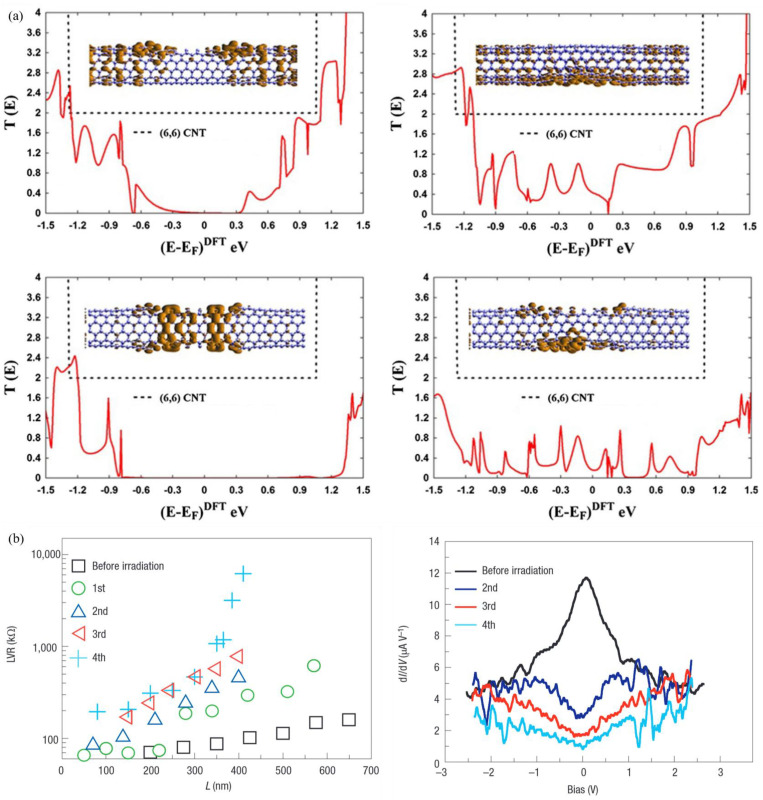
(**a**) The zero bias transmission probabilities with different defect types [114]; (**b**) effect of consecutive irradiations on the electrical resistance [115].

The application of density functional theory in conjunction with Green’s function scattering approach facilitates the elucidation of defects’ impact on electronic transport properties. The conductance of defective carbon nanotubes (CNTs) has been shown to typically exhibit non-trivial behavior, which has been observed to decrease and affect the band gap. In more precise terms, elevated levels of disorder have been shown to induce a greater degree of localization of DOS (Figure 8a) [114].

Furthermore, experimental results can be obtained. Navarro et al. [115] applied consecutive Ar^+^ irradiation doses to SWCNTs in order to produce a uniform density of defects. The data demonstrate an exponential dependence of resistance on SWCNT length, thus indicating that the system is within the strong Anderson localization regime (Figure 8b).

A number of strategies have been developed for the purpose of defect healing to restore the conductivity, such as thermal annealing. Khanbolouki et al. [116] applied current-induced annealing (5–60 MA/m^2^ in high vacuum) at 685–1445 °C to remove impurities, which results in the reduction of sheet resistance by 35–40%. Altuntas et al. [117] found that annealing at 120 °C for 1 h in air led to a reduction in interface traps and contact resistance, from 1.9 MΩ to 12.3 kΩ.

### 3.2. Electrical Properties of Several CNTs

It has been demonstrated that due to van der Waals interactions, individual SWCNTs, which are nearly uniform in diameter, readily self-organize into “ropes” [118]. A more common statement is CNT bundles, which also represent the intermediate transitional form from individual tubes to macroscopic CNT assemblies. Consequently, considerable research has been devoted to understanding how the interaction in bundles affects the electrical properties of the CNT assemblies. In this section, an exploration of the primary limitations on electrical properties in multi-tube systems, comprising two CNTs and CNT bundles, will be conducted.

#### 3.2.1. Electrical Properties of Two CNTs

The density functional tight (DFT) binding theory and the nonequilibrium Green’s functions are frequently utilized to calculate the electrical properties of pairs of CNTs. The template structures utilized in the calculation for two CNTs can be categorized into three distinct types: tip-to-tip, parallel, and perpendicular. Among these studies, two parallel armchair SWCNTs with equal chirality indices are always considered first, as they are the simplest model. However, experimental measurements across pairs of CNTs are much more difficult due to the nanomanipulation involved in SEM or TEM. To date, experimental measurement of junction resistance has been possible only for two MWCNTs.

**Figure 9:**
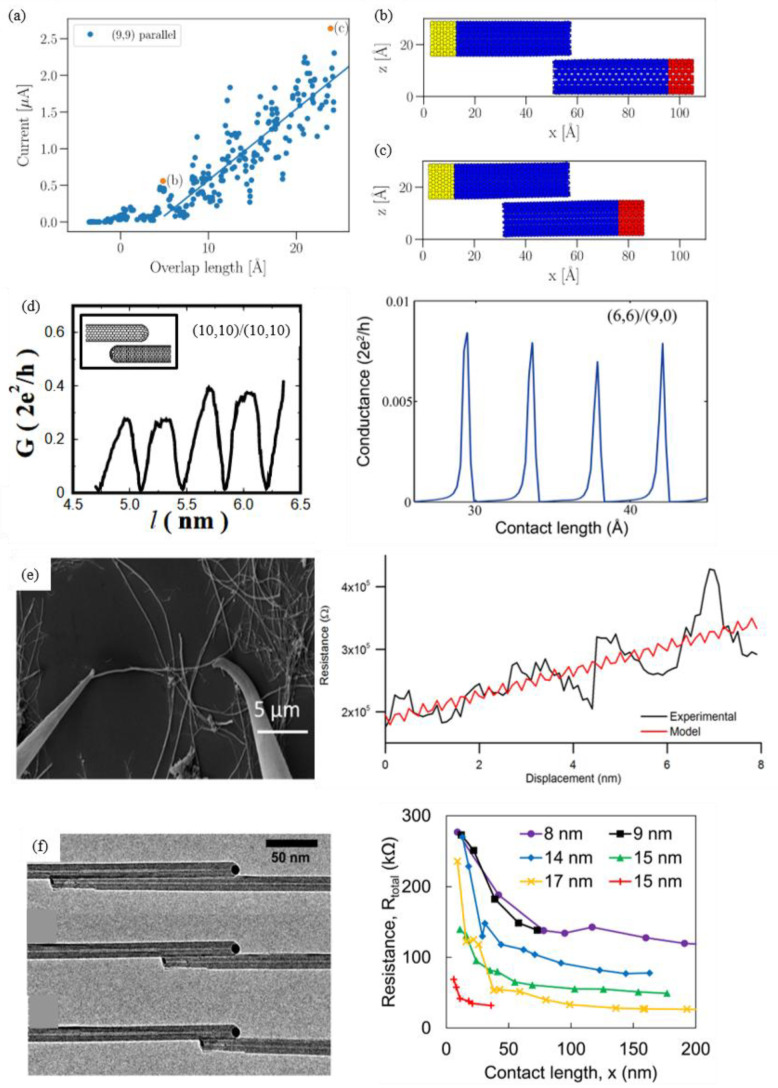
(**a**) Relationship between overlap length and current for pairs of parallel (9,9) SWCNTs [119]. (**b**,**c**) Geometries of two highlighted points in orange [119]. (**d**) The calculation results for the variation in conductance at the Fermi energy as a function of overlap length for two (10,10) SWCNTs [123] and a (6,6)/(9,0) contact [124]. (**e**) The SEM image and the schematic of manipulating two halves of a same MWCNT in a parallel manner. The bottom plot is the measured resistance at 1V as a function of the overlap length, where the red line is a simple model result [122]. (**f**) In situ TEM image of two parallel MWCNTs with various contact lengths. The right plot is the electrical resistance as a function of contact length for different MWCNTs. Legend indicates the outer diameter of each MWCNT [45].

The following discussion will commence with a consideration of two CNTs arranged in parallel. It has been demonstrated that there is a positive correlation between the overlap length and the electrical current (Figure 9). As demonstrated in Figure 9a, the scatter plot of the calculation results indicates that an overlap length of two armchair SWCNTs in parallel greater than 25 Å can induce a larger current [119]. A more detailed analysis of the results is presented in Figure 9d, where nanotube junctions are formed by two (10,10) SWNCTs and (6,6)/(9,0) SWCNTs. A noteworthy observation is the occurrence of a nonlinear and quasi-periodic relationship between conductance and contact length. These quasi-periods were found to be heavily dependent on the chirality of SWCNTs. For instance, armchair SWCNT and zigzag SWCNT are about 3a_0_ and a_0_, respectively. This is due to the fact that the Fermi wavelength for armchair tubes is a_0_, where a_0_ is the unit cell parameter [111,120]. When contact lengths are compared, the conductance of the armchair/zigzag contact is found to be significantly lower than that of the armchair/armchair contact, by approximately one order of magnitude. The reason for this phenomenon is that two SWCNTs with different chirality are incommensurate and possess mismatched Fermi momenta [121]. It can be concluded that the optimal conductance at a parallel contact can only be achieved when two metallic tubes possess the same chirality. Examples of such chirality include metallic zigzag/zigzag and armchair/armchair types. It is evident that the metallic zigzag tubes possess a gap around the Fermi energy due to the curvature effect. This observation indicates that armchair/armchair tubes in parallel are the most promising geometry for connecting multiple tubes in device applications [111,120].

This similar periodical trend was experimentally observed by Barnett et al., in which two halves of the same MWCNTs were manipulated in a SEM (Figure 9e) [122]. There is an apparent periodicity of 2–3 Å, consistent with the unit cell of armchair SWCNT (2.45 Å). In addition, Hamasaki et al. also reported the in situ measurement of the parallel-contacted resistance between two halves of the same MWCNT in TEM [45]. The hypothesis was that the contact resistance would decrease with increasing contact length within the measurement range of a few hundred nanometers. It is theorized that, under certain conditions, the effect of the interface may become negligible, provided that the contact length is sufficiently long.

**Figure 10:**
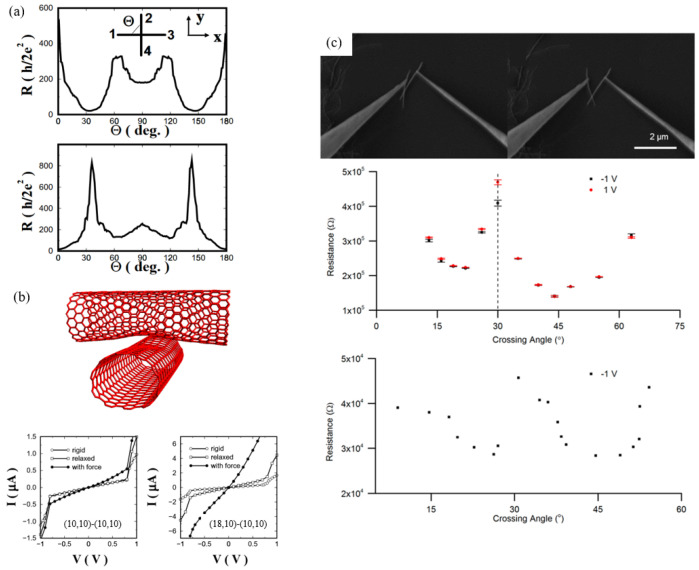
(**a**) The variation in contact resistance as a function of the rotation angle [123]. (**b**) The schematic of the cross-junction where the two SWCNTs are perpendicular to each other. I-V characteristics of a (10,10)–(10,10) (out-of-registry) and (18,10)–(10,10) (in-registry) cross-junctions [123]. (**c**) SEM images of manipulating the two halves of an individual MWCNT with different crossing angles. Plots of resistance at ±1 V as a function of junction angle with two different MWCNTs [122].

Due to the occurrence of angular overlap conduction, the conductive rule is analogous. When one SWCNT is placed on top of another, the conductance between the in-registry tubes is increased. In this instance, the contact region is commensurate, with the carbon atoms arranged in an A-B stacking configuration of graphite [123]. The plot in Figure 10a predicts the resistance variation concerning the rotation angle along the tube axes by calculation. The tubes in the first (18,0)–(10,10) junction are found to be in registry at θ = 30, 90, 150°. The tubes in the second (10,10)–(10,10) junction are arranged in registry at θ = 0, 60, 120, 180°. Lower resistances have been observed in these in-registry orientations. However, even when the tubes are in registry, the low resistance can vary at different rotation angles. The lowest possible resistance is achieved when the carbon atoms in contact area are stacked like A-A stacking of graphite. This variation in electrical properties bears a resemblance to the dependence of mechanical or frictional properties on atomic registry [125,126]. The variation in resistance as a function of the crossing angle between two unparallel tubes is also investigated. As the crossing angle is reduced, the contact resistance decreases because the contact area is reduced; however, for MWCNTs with large diameter and low weight, the change in contact area will be negligible. Barnett et al. conducted a study on the angular dependence of contact resistance of two halves of a single MWCNT in in situ SEM (Figure 10c). The findings demonstrate that the two tubes manifest as “in-registry” at 22° and 44°, exhibiting reduced resistance. This observation signifies that the concept of “in-registry” for MWCNTs is less stringent than the ideal SWCNT. It is conceivable that the minima occur when the overlap of the π-orbital or electrostatic forces is minimal, leading to reduced repulsion [122].

Furthermore, irrespective of the commensurate status of the contact area, the electronic contact can undergo significant enhancement through the application of forces and relaxation (Figure 10b). However, the mechanism of the transport properties of two CNTs remains unclear because of the lower precision for atomic manipulation and characterization. Further study is required to gain a more profound comprehension of the mechanisms.

#### 3.2.2. Electrical Properties of CNT Bundles

As Thess et al. [118] initially demonstrated, SWCNTs have the capacity to self-assemble with remarkable uniformity into crystalline ropes, which are more commonly referred to as bundles. The electrical properties of CNT bundles are governed by their mixed fine structures, weak inter-tube coupling, and low-dimensional quantum effects.

Bockrath et al. [127] revealed the profound impact of this heterogeneity at low temperatures. Measurements on individual bundles demonstrated a suppression of conductance at low bias (<10 K) and sharp peaks in conductance as a function of gate voltage. These features were interpreted as single-electron charging and resonant tunneling through quantized energy levels within the bundle. Collins et al. [120] addressed the challenge of exploiting s-SWNTs within mixed bundles by introducing “electrical breakdown”. The exploitation of high current stress in air, which selectively destroys only the m-SWCNTs within the bundle, results in the conversion of the entire bundle into an effective FET based solely on the remaining s-SWCNTs.

The core limitation for CNT bundle conductivity is inefficient electron transfer at inter-tube junctions. This phenomenon was clearly illustrated through the use of quadrupole STM on herringbone-type CNTs [128]. The junctions were found to adopt a V-, T- and Y-shaped configuration, thereby functioning as ohmic scattering centers. However, they exhibited no gating effect, a phenomenon that can be attributed to the strong inter-layer coupling effect, which effectively suppresses electrostatic modulation.

A number of strategies have been proposed to improve inter-tube coupling. Ha et al. [129] employed laser-induced shockwaves (~3.2 GPa) to densify CNT networks. This process physically transformed CNT bundles into flattened, multi-layered graphene nanoribbons. The combination of pre-stretching for alignment and the other factors under investigation resulted in an approximate fivefold increase in electrical conductivity. This is due to the elimination of voids and the creation of seamless pathways for electron transport via graphitized interfaces. Gong et al. [130] utilized in-situ TEM to form covalent graphitic bonds through Joule-heating-induced welding, thereby augmenting bundle conductivity from approximately 10^2^ S/m to 10^5^ S/m. Qiu et al. [131] employed a two-step strategy, namely interfacial functionalization and densification, to increase the inter-bundle electrical properties. Infiltration with high polarity solvents such as ethylene glycol induced local electrostatic cohesion between bundles via surface dipoles, increasing interfacial electrical conductance by approximately 2.8 times. Acid treatment (HNO_3_) introduced oxygen-containing functional groups acting as electron relays. This resulted in an additional 95% increase in inter-bundle electrical conductance.

### 3.3. Electrical Properties of Macroscopic Assemblies of CNTs

The assembly of CNTs into various macroscopic structures, such as fibers, arrays, and cottons, is contingent upon the specific synthesis process employed. Under these conditions, individual CNTs and CNT bundles agglomerate due to van der Waals interactions. Consequently, introducing inter-tube interfaces and non-uniformity of CNTs has resulted in a challenging task for preserving the unique electrical properties of individual CNTs in CNT macrostructures. In this section, the issue of dependence on the electrical properties of the assemblies of CNTs, such as fibers and films, will be explored in depth. The existing methods for improving their electrical properties will also be employed.

#### 3.3.1. Electrical Properties of CNT Fibers

**Figure 11:**
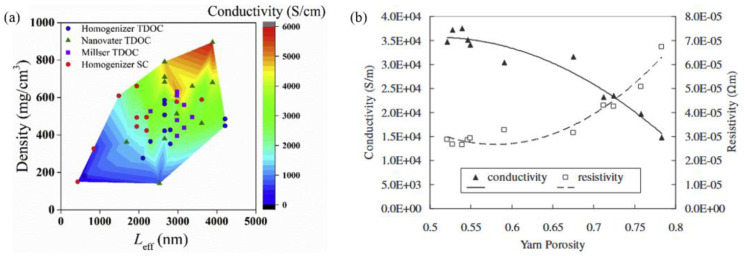
(**a**) Contour plot of electrical conductivity as functions of the effective length and fiber density [135]. (**b**) Variation in conductivity and resistivity as a function of the yarn porosity [136].

The electrical properties of CNT fibers are contingent on the intrinsic structure of CNTs present within the macrostructure, including chirality, diameter, and length. Additionally, the extrinsic configuration, such as porosity and the alignment of CNT fibers, must be also considered (Figure 11).

In order to achieve an optimal electrical conductivity, the CNT fibers must consist exclusively of defect-free armchair SWCNTs, characterized by their small diameter, long length, and single chirality. As Sundaram et al. [132] reported, the electrical conductivity of fibers spun from metallic CNTs is expected to be high. The quantity of shells has been demonstrated to influence the conductivity of individual CNTs, as well as the collective conductivity of the fiber. The conductivity of SWCNTs is greater than that of MWCNTs because the mean free path decreases with an increase in the number of walls [133]. It can be posited that even in the context of purely armchair MWCNT fiber, the formation of physical contact between the internal shells and other nanotubes is improbable. Therefore, perfect electronic coupling between the inner and outer shells may prove challenging to achieve [134]. Tajima et al. [135] demonstrated that the specific conductivities of the fibers are almost proportional to the effective length of CNT. It is imperative to note that the increased length of the carbon nanotubes (CNTs) within the fiber will ensure a sufficient number of inter-tube contacts during the passage of a charge along the fiber [134].

It is noteworthy that extrinsic configuration substantially influences the electrical conductivity of CNT fibers. It is hypothesized that if the CNTs in a fiber are well condensed and perfectly axially aligned, it will ensure a long contact area with good physical stability and the shortest pathway for the flow of charge. This will result in a decrease in the overall resistance of the fibers. Miao et al. [136] systematically studied the relationship between the conductivity and the porosity of the CNT fibers. The study’s results demonstrated a decreasing conductivity trend with increasing porosity. Francis et al. [137] theoretically suggested that the peak electrical sensitivity is attained within 40% to 60% porosity range. Furthermore, it has been demonstrated that enhancing the degree of alignment represents an effective strategy for enhancing the electrical conductivity and capacity of CNT fibers. The increase in alignment can be achieved through the application of outfield, stretching [138], twisting [139], or a combination of gravity and drawing [52], amongst other methods.

#### 3.3.2. Electrical Properties of CNT Film

**Figure 12:**
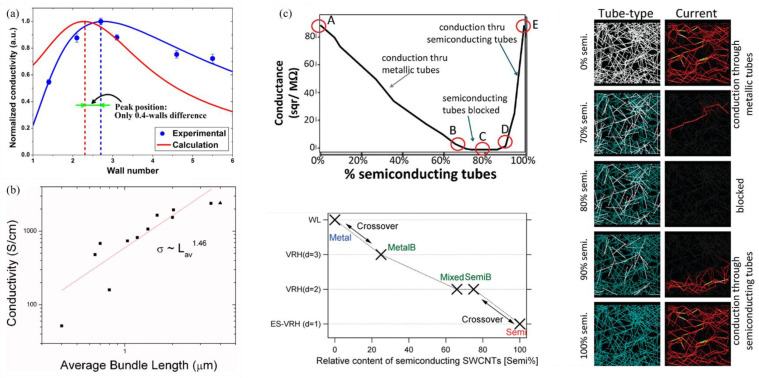
(**a**) The variation in calculated electrical conductivity in red line and normalized experimental conductivity in blue line as a function of wall number [110]. (**b**) The relationship between the average bundle length and electrical conductivity of CNT network [162]. (**c**) Electronic phase diagram of conduction mechanisms as a function of semiconducting tube ratio [146,148].

The primary factor influencing the electrical performance of CNT transparent conductive film (TCF) is the microstructure of CNTs. It has been demonstrated that long CNTs (about 10–20 μm) [140] with a large diameter (the diameter of the SWCNT and DWCNT is about 1.4 nm and 4nm, respectively) [141,142] and high crystallinity [143] are preferable for the construction of high-performance TCFs. The wall number is also crucial for the performance of TCF. It is evident that SWCNT film is the most desirable material for electronic and optoelectronic devices, given that it contains numerous semiconductive tubes and MWCNT absorbs more photons (Figure 12a) [110,142,144]. Furthermore, the incorporation of DWCNTs with a large diameter (about 4 nm) has been demonstrated to result in a substantial enhancement of the yield threshold of bundling, thereby expanding the contact area at the junctions [142]. The electrical type is also a crucial factor in this regard [145,146,147,148]. In a typical SWCNT film, the existence of Schottky barriers between metallic and semiconducting tubes invariably results in the presence of hopping barriers, thereby preventing high conductivity. As revealed by Yanagi et al. [148], quantum transport can be observed and hopping barriers are not present in SWCNT networks formed by high-purity metallic tubes, while a Coulomb-gap-type conduction mode is observed in semiconductive SWCNT networks. Topinka et al. and Yanagi et al. [146,148] provided a summary of the conduction mechanism in SWCNT networks as a function of the metallic/semiconducting ratio (Figure 12c). As the relative content of semiconducting SWCNTs increased, the conduction mechanism underwent a transition from quantum transport to variable range hopping (VRH) and then from VRH to ES-VRH. The VRH model postulated strong localization of electrons, indicating strong disorder in the contact area between bundles. The ES-VRH model indicated the presence of Coulomb interactions between localized electrons, inducing a Coulomb gap in the DOS. It was observed that both m-SWCNT and s-SWCNT networks exhibited the highest conductivity.

It is also imperative to optimize the configuration of CNT networks to enhance the electrical performance of TCFs. Many experiments have revealed that TCF consisting of isolated CNTs exhibits superior conductive properties compared to bundles [149,150,151,152]. Moreover, to avoid the introduction of surfactants and defects, it is preferable to synthesize an isolated CNT network by FCCVD directly [132,135] instead of utilizing solution-based techniques [61,153,154,155]. For instance, Kauppinen et al. [156] obtained SWCNT networks with over 60% isolated tubes by FCCVD method. After a HNO3 doping process, the TCFs demonstrated a comparatively low sheet resistance (R_s_) of approximately 63 Ω/sq at a transmittance of 90% for 550 nm light. The synthesis of carbon-welded SWCNT film composed of approximately 85% isolated tubes was achieved by Liu et al. using the injection FCCVD method [149]. For pristine and HNO_3_-doped TCFs, the Rs values were recorded as 41 Ω/sq and 25 Ω/sq, respectively. The high concentration of isolated CNTs in TCF is crucial to its ultrahigh performance. This is due to the provision of sufficient carrier transport paths without the absorption of additional light [145].

The percolation theory is widely used to examine the transport behavior of TCFs [157,158,159], which is employed to delineate the insulator-to-conductor transition. The percolation threshold $N_{C}$ is the minimum CNT concentration at which a conductive pathway can be formed, as illustrated in Formula (3).

(3) $$N_{C}=4.236^{2}\pi L_{s}^{2}\approx \frac{1}{L}$$

*L_s_* and *L* represent an individual CNT length and aspect ratio, respectively. This indicates that the percolation threshold for SWCNT films with large aspect ratios is low, indicating that a minimal surface coverage is required to establish carrier transport pathways. Concurrently, the film resistance undergoes a precipitous decline above the threshold at elevated CNT densities. In this case, CNT hybrid architectures have been shown to synergize aligned domains (providing directional strength, conductivity, and deformation resistance) with random networks (delivering isotropic resilience, stress absorption, and environmental buffering) to mitigate anisotropy limitations. This balance optimizes mechanical stability, enhances charge transport via directional pathways and percolation networks, and improves environmental adaptability—particularly against humidity or temperature shifts. Hu et al. [160] derived a power exponential relationship between film conductance and thickness by controlling the volume of CNT suspension to form the TCF by vacuum filtration. Furthermore, a reduction in the size of the tube bundle results in a decrease in its square resistance [161]. It has been demonstrated by experimental and theoretical works that the network conductivity of CNTs is approximately linearly related to the mean bundle length and the network thickness, and approximately related to the 0.5 power of the mean bundle diameter (Figure 12b) [162].

The degree of alignment is another factor that affects the conductivity of TCFs, and the cutoff angle (θ_μ_) is often used to quantify the degree of alignment. If the degree of alignment is excessive or insufficient, the formation of a conductive percolation path is impeded due to anisotropy or inadequate coverage, respectively. In contradistinction, the conductivity of CNT films is elevated by random distribution, a phenomenon attributable to diminished percolation thresholds [163,164]. Furthermore, the presence of amorphous carbon and sp^3^ bonds within the network leads to carrier scattering, demonstrating that films with a high G/D ratio exhibit enhanced conductivity [165].

**Figure 13:**
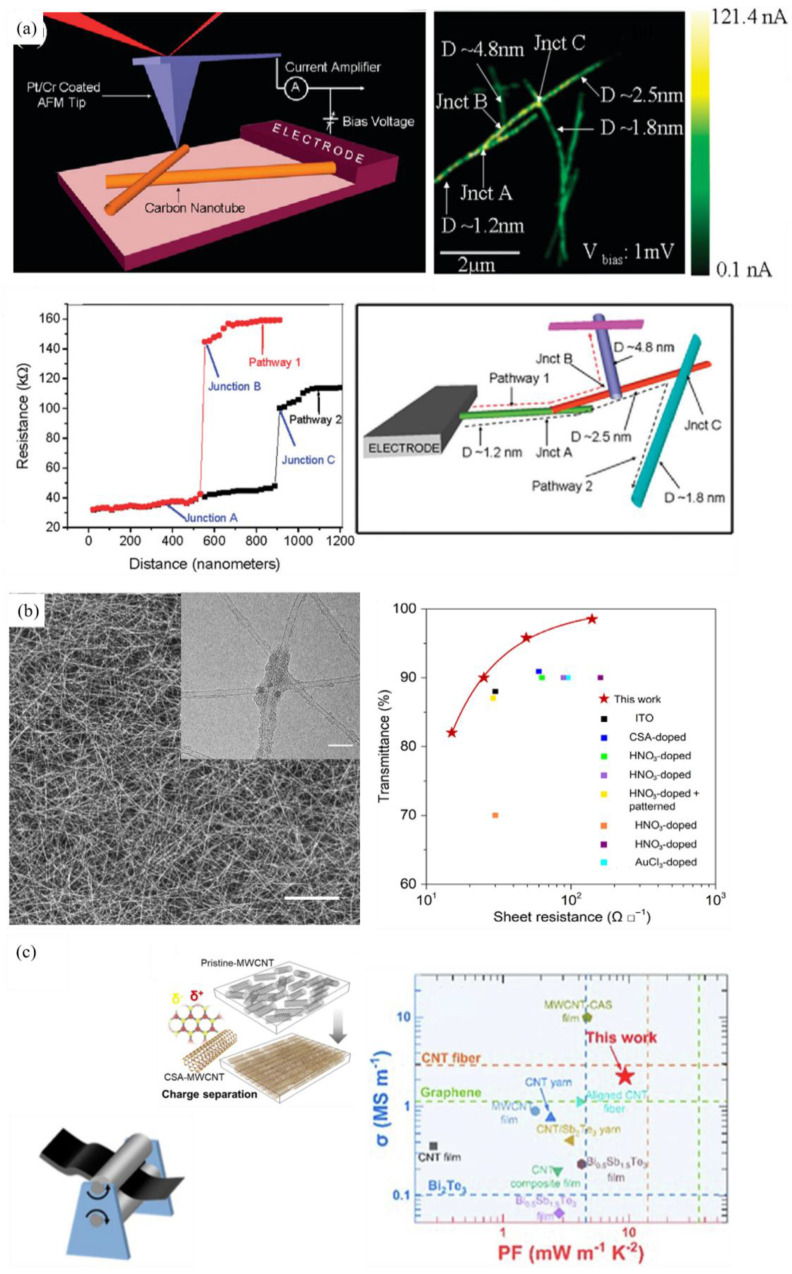
(**a**) Schematic of conductive mode in atomic force microscope, and one of the current maps and resistance analysis of interconnected tubes [147]. (**b**) The left are the SEM and TEM images of SWCNTs with carbon-welded joints. The right is the transmittance (550 nm light) versus Rs of doped SWCNT TCFs and other reported doping results [149]. (**c**) Schematic of compression and acid treatment for densification, SEM images of pristine and compressed CNT films, and the thermal and electrical performance compared to various materials [157,158].

It is widely accepted that the junction resistance (Figure 13a) [147] is the primary contributor to the Rs of a TCF, suggesting that enhancing the performance of TCFs is more effectively achieved by focusing on the control of inter-tube connections rather than the structure of composing CNTs. A considerable amount of effort has been invested into the enhancement of junction resistance. The integration of graphitic carbon within the junctions has been demonstrated to facilitate a transformation of the Schottky contacts between s-SWCNT and m-SWCNT, converting these contacts into near-ohmic configurations (Figure 13b) [149]. Also, controlling the unique morphology of the network, characterized by a preponderance of Y-junctions over X-junctions, which possess larger contact areas, has the potential to yield reduced junction resistances [61]. Moreover, densification through the application of acid treatments, such as HNO_3_ [166] and chlorosulfonic acid (CSA) [167] or employing simple compression [166,167,168] could also enhance the conductivity of TCFs. It enhances the zipping effect between tubes, leading to highly packed and oriented CNTs in the film, with a concomitant decrease in voids (Figure 13c).

### 3.4. Discrepancy in the Electrical Property of Individual CNTs and Their Assemblies

It has been demonstrated that individual CNTs, especially individual SWCNTs, exhibit excellent electrical properties. The conductivities of SWCNTs can be comparable to, or even superior to those of highly conductive metals. In an ideal condition, the carrier mobility and current-carrying capacity of a SWCNT with a defect-free structure can be extremely high. However, when assembled into macrostructures, the distinct transport properties of the individual tubes do not translate into the assemblies. Table 2 summarizes experimental results of the electrical properties of individual CNTs and their assemblies.

The significant difference in the electrical properties of individual CNTs and their assemblies can be attributed to the following factors: First and foremost, is the cross junctions exhibited in assemblies. Since the inter-tube contact at cross junctions is achieved by van der Waals interaction, it may be difficult to achieve an ohmic contact. The second factor is a mixture of nanotubes that vary in chirality, diameter, number of walls, and length in the assemblies. It is still challenging to prepare pure SWCNTs with identical structures. Thirdly, some overall structural factors like tube alignment, densification, and porosity also significantly influence the properties. If the arrangement is highly disordered, the electron transport paths become zigzag, which leads to a decrease in the conductivity.

As a result, a large number of strategies for optimizing the electrical performance of assemblies have been proposed. On orientation control, CNTs are arranged by drawing [52,169], applying electric field [170,171], or using template method [172] to reduce the interface resistance. Substitutional or surface charge transfer doping was also performed to improve the electrical conductivity (Section 3.5). Additionally, in order to obtain reliable conductivity measurements, it is necessary to undertake pre-treatments like acid treatment to remove residual impurities and post-treatments like high-temperature annealing to reduce defects. It should also be noted that trade-offs between mechanical and electrical properties invariably occur in CNT assemblies. Increasing the density and alignment of CNTs improves the electrical properties and also promotes the mechanical properties by improving the interfacial resistance. However, certain methods, such as high-temperature treatment, have been observed to enhance electrical properties at the expense of toughness due to reduced inter-tube overlap [173].

### 3.5. Electrical Property of Doped CNTs

As mentioned above, the electrical properties of individual CNTs are strongly dependent on their atomic structures, such as chirality and defects. In contrast, the electrical properties of assemblies are also influenced by inter-tube junctions, extrinsic configurations such as porosity, and alignment. A number of strategies are proposed to enhance the conductivity in both Section 3.2 and Section 3.3. However, it is worth noting that the atomically controlled growth of CNTs and charge transfers in CNT assemblies have yet to be fully achieved. Doping is a critical strategy for tailoring the electrical properties of CNTs and has broad application prospects in electronic devices. There are mainly two primary doping methods: substitutional doping and surface charge transfer doping.

#### 3.5.1. Substitutional Doping

Substitutional doping in CNTs incorporates foreign atoms into the carbon lattice. This is similar to the bulk doping process used in conventional semiconductors. Owing to their similar atomic radii to carbon (C) atoms, nitrogen (N) [174,175,176,177,178] and boron (B) [179,180,181] are the most commonly used doping elements, which release excess electrons to CNTs and create holes in the valence band in CNTs, respectively.

There are basically two approaches to prepare the substitutionally doped CNT: in situ doping during synthesis and post-synthesis doping. For the former, precursors containing the dopant atoms are used during CNT growth, by arc discharge [182,183], laser ablation [184,185,186], chemical vapor deposition (CVD) [187,188], spray-pyrolysis [177,189], or plasma-assisted CVD [190]. For the later, post-synthesis chemical reaction was performed to replace carbon atoms in CNTs with doping atoms, which can be realized by heat treatment [191,192], plasma treatment [193,194], or ball-milling [195,196].

B-doped and N-doped CNTs exhibit unique electronic and transport properties. At low concentrations of B or N, the dopants are uniformly distributed within the CNT and the impurity states appear in the band gap, which can be detected by STM. The donor state induced by N doping appears near the conduction band minimum, while the acceptor state in the case of B is close to the valence band maximum. Moreover, some calculation results suggested that the localization of the extra charge associated with the impurity differs considerably between s-CNTs and m-CNTs. Compared to an s-CNT, the defect state is spatially more localized for a metallic one, resulting in a significant difference in electrical and chemical properties [197].

With a high concentration of B or N, several studies have revealed that N-doped and B-doped CNTs exhibit metal-like behavior [198] because of the presence of dopant-rich islands like BC_3_ nanodomains [199] and CN_x_ tubes [200].

Owing to their excellent conductivity and chemical stability along with unique electronic structure and high electron density, N-doped and B-doped CNTs have been widely used in flexible micro-supercapacitors, batteries, and catalysis.

#### 3.5.2. Surface Charge Transfer Doping

Surface charge transfer doping is an unconventional way to achieve doping through surface engineering, which is commonly used in the doping of CNT assemblies. Take p-type doping as an example, acceptors are known to possess unoccupied molecular orbitals for electrons (UMOs). In the event of the energetically lowest of these orbitals (LUMO) being in proximity to the valence band maximum of the CNT, an electron will be transferred from the CNT. Consequently, holes will form in the CNTs, and negative charge will be localized on the surface acceptors. The process of charge separation is accompanied by the establishment of an electrostatic potential that confines the holes in a perpendicular direction. However, the holes are still able to move in a parallel direction to the surface [201].

Ionic dopants form a heterogeneous interface between CNT and dopants after CNT synthesis. N-type dopants like alkali metals (Li [202], K [95,203,204,205,206,207,208]) and p-type dopants like halogen elements (Br_2_ [208], I_2_ [209,210,211,212,213]), strong acid (CSA [167,209]), and transition metal halides (CuI [214], Ibr [209,215], Icl [215], AuCl_3_ [216,217], FeCl_3_ [203,218], PbI_2_ [219]) are the most commonly used dopants. Regarding the location of the dopants, the doping type can be classified as exohedral (intercalation and deposition) and endohedral (filling) [220].

**Figure 14:**
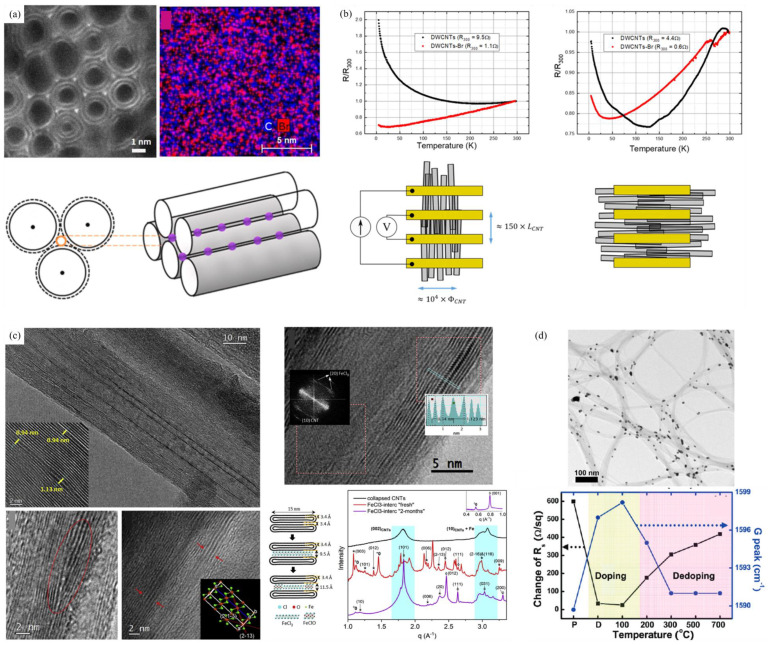
(**a**) High-angle annular dark-field (HAADF)–scanning transmission electron microscopy (STEM) image, energy dispersive X-ray chemical map, and illustrations of the interstitial doping of the cross section of a Br-intercalated DWCNT fiber [221]. (**b**) Low-temperature transport properties before and after Br-intercalation with different transport directions [221]. (**c**) High-resolution TEM image of FeCl_3_-intercalated collapsed CNT fiber and evidence of the existence of FeClO and FeCl_3_ [222]. (**d**) TEM image of AuCl_3_-doping SWCNT film and the variation of Rs and G peak position as a function of temperature [217].

A substantial body of research remains on exohedral doping (Figure 14). Madrona et al. [221] demonstrated that the intercalation of long-range ordered Br into DWCNT fibers reduces the inter-tube resistance, and increases the conductance of individual DWCNTs. Madrona et al. [222] introduced FeCl_3_ intercalation into fibers consisted of collapsed CNTs, which leads to a stable increase in conductivity measured at a factor of six. Qiu et al. [223] deposited Au nanoparticles in CNT fibers to enhance the interfacial electrical transport, inducing an equivalent p-type doping.

**Figure 15:**
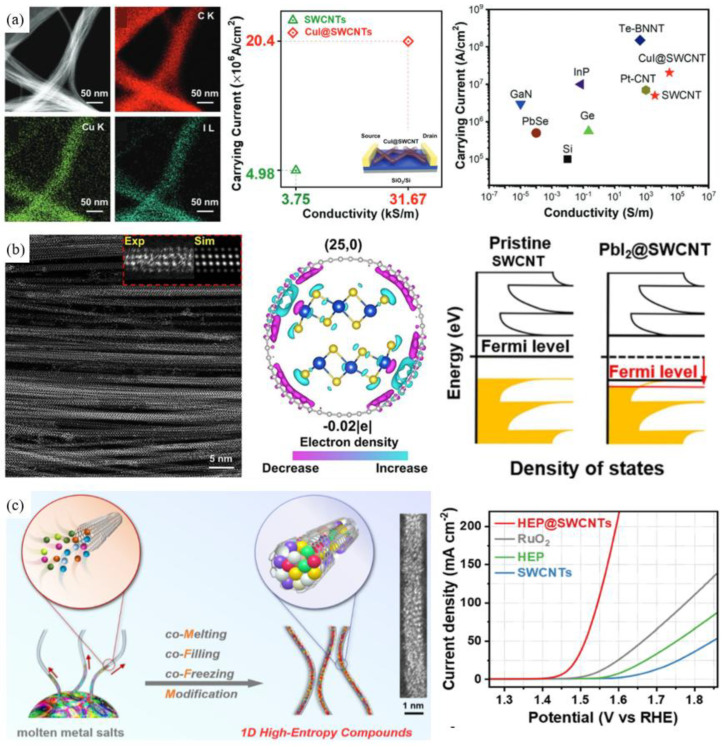
(**a**) Elemental mapping images and electrical performance of CuI@SWCNT networks [224]. (**b**) HAADF STEM image, structural models, DFT calculations showing the electron density difference and Scheme showing the downshift of the Fermi level of PbI_2_@SWCNT [219]. (**c**) Schematic illustration for the synthesis and the electrochemical characterization of HEP@SWCNTs [225].

Recently, considerable attention has been paid to the construction of one-dimensional van der Waals heterostructures by endohedral doping for the purpose of enhancing the electrical properties. Due to the confinement protection of CNTs, they exhibit good stability. Zhang et al. [224] prepared CuI@SWCNT networks with an electrical conductivity of 32 kS m^−1^ and a current carrying capacity of 2 × 10^7^ A cm^−2^ (Figure 15a). Teng et al. [219] prepared a PbI_2_@SWCNTs hybrid and fabricated self-powered photodetectors that exhibit exceptional photocurrent and a switching ratio of three orders of magnitude (Figure 15b). Du et al. [225] developed a synthesizing method named comelting–filling–freezing–modification for simultaneously encapsulating various high-entropy metal phosphide (HEP) into SWCNTs. The SWCNT could protect the 1D HEP and donate π electrons to the HEP for enhanced electron delocalization, thus promoting high electrocatalytic activity and stability (Figure 15c).

It is important to note that the substitutional doping always demonstrates superior thermal and chemical stability compared to the surface charge transfer doping because the substitutional dopants exhibit superior thermal resilience via strong chemical bonds. The dopants employed in surface charge transfer doping, instead, are susceptible to de-doping in high temperature or humid/ionic environments. This limitation restricts the applicability of this method to high-temperature applications like cables.

## 4. Conclusions and Outlook

In summary, CNTs are a promising candidate for use in electronics, energy storage, transparent conductive films, and other electrical applications due to their exceptional electrical properties. This review briefly introduces the methods explored for measuring the electrical properties of CNTs, such as STM, electron microscope-based nanoprobes, CNT electronic devices. These methodologies can be used to obtain a variety of electrical information, such as electronic band structure, resistance, conductance, and thermoelectric power. Most approaches require contact between CNTs and electrodes, inevitably leading to measurement errors. In addition to contact resistance, other factors such as measurement environment and substrate have also been identified as contributors to the diversity of electrical measurement results in different experimental groups. Moreover, the effects of intrinsic structures of CNTs such as chirality, diameter, defects, and length on the electrical properties of individual CNTs at the atomic scale are discussed. More importantly, the primary limitations on electrical properties in multi-tube systems have been explored by considering two CNTs, CNT bundles, fibers, and films. The core limitations for charge transfer are clarified as inter-tube junctions and extrinsic configurations of CNT assemblies, such as porosity, alignment, etc. Several strategies such as acid treatment, application of outfields and doping have been developed to modify the alignment, van der Waals interactions, and contact resistance of CNTs in macroscopic assemblies.

Despite the development made in improving the electrical properties of individual CNTs and CNT assemblies, many challenges still remain. Firstly, the electrical transfer mechanism from individuals to assemblies remains unclear. Although a general understanding is that the inter-tube interfaces is the main origin of the degraded electrical properties, there has been no general theory, principle, and method to solve this problem. Secondly, bridging the gap between theory and experiment is still challenging. The structure of CNTs cannot be precisely controlled and measurement errors do exist.

Based on the current research status and the aforementioned challenges, we propose the following strategies to address these issues: First of all, defects are inevitably introduced during CNT synthesis and subsequent processing, and these defects act as scattering centers of electron transport. It is important to develop defect engineering techniques, such as laser annealing and chemical functionalization, which enables vacancy healing in a gentler and more controllable way. Secondly, the transport properties of CNTs are strongly dependent on their structures, especially their chirality. Consequently, realizing chirality-specific synthesis is an important way to boost the electrical properties of individual and assembled SWCNTs. Thirdly, advanced doping methods need developed to achieve more stable n/p-type CNT assemblies. Fourthly, modifying the interface structure between CNTs in their macroscopic assemblies is highly important. It is expected that efficient electrical conduction will be realized through the optimization of the assembly configuration and the interaction between the comprising CNTs. The application of machine learning to guide assembly design [226] and conduct high-throughput synthesis screening for CNT assembly optimization [227,228] is also a viable option.

## Figures and Tables

**Table 1:** A comparison of the methods for reducing the contact resistance at individual CNT–electrode interface.

Method	Advantage	Disadvantage
High-temperature annealing [89]	Batch processing Forming stable low resistance carbides	Large high-temperature zone
Local Joule heating [90]	Easy to operate Highly targeted treatment	Poor repeatability
Electron beam-induced deposition [91]	Reliable and good electrical contact	Harsh condition Low efficiency
Electron beam irradiation [42]	Effective improvement in electrical contact	Small processing area Damage to CNTs
Ultrasonic nanowelding [92]	Fast and reliable, normal temperature operation, wide range of adaptation	Difficulty in precise control

**Table 2:** Experimental result of the electrical properties of individual CNTs and their assemblies.

	Individual CNTs	Assemblies of CNTs
Conductivity	10^6^–10^7^ S/m (m-SWCNT) 10^2^–10^3^ S/m (s-SWCNT)	10^3^–10^5^ S/m
Carrier mobility	10^5^ cm^2^/(V·s) (m-SWCNT) 10^4^ cm^2^/(V·s) (s-SWCNT)	~10^3^ cm^2^/(V·s)
Current carrying capacity	~10^9^ A/cm^2^	~10^6^–10^7^ A/cm^2^
Anisotropy	One-dimensional(1D) conductor Extremely anisotropic	Alignment CNT: anisotropic Disordered CNT: ~isotropy
Stability and environmental sensitivity	Susceptible to surface adsorbents (such as oxygen, water), resulting in electrical performance fluctuations	Higher structural stability, but the interface oxidation or mechanical deformation may occur for a long time.
Application	Nanoelectronic devices (transistors, sensors), quantum wires, scanning probes, etc.	Flexible conductors, electromagnetic shielding materials, battery electrodes, composite reinforcement phase

## Abbreviations

The following abbreviations are used in this manuscript:

CNTs	Carbon nanotubes
SWCNTs	Single-walled carbon nanotubes
m-SWCNTs	Metallic single-walled carbon nanotubes
s-SWCNTs	Semiconducting single-walled carbon nanotubes
DWCNTs	Double-walled carbon nanotubes
MWCNTs	Multi-walled carbon nanotubes
FET	Field-effect transistor
STM	Scanning tunnelling microscopy
STS	Scanning tunneling spectroscopy
DOS	Density of states
SEM	Scanning electron microscope
TEM	Transmission electron microscope
FIB	Focused ion beam
ICs	Integrated circuits
MOSFET	Metal–oxide–semiconductor field-effect transistor
FCCVD	Floating catalyst chemical vapor deposition
TCF	Transparent conductive film
VRH	Variable range hopping
CSA	Chlorosulfonic acid
1D	One-dimensional
HAADF	High-angle annular dark-field
STEM	Scanning transmission electron microscopy
HEP	High-entropy metal phosphide
DFT	Density functional tight
